# Patient health engagement as a moderator between perceived stress and treatment adherence among kidney failure patients undergoing hemodialysis: a cross-sectional analysis

**DOI:** 10.1007/s10865-025-00591-w

**Published:** 2025-07-23

**Authors:** Dilara Usta, Marta Acampora, Guendalina Graffigna

**Affiliations:** 1 EngageMinds HUB– Consumer, Food and Health Engagement Research Center, Catholic University of Sacred Heart, Cremona, 26100 Italy; 2 Faculty of Agriculture, Food and Environmental Sciences, Catholic University of Sacred Heart, Cremona, 26100 Italy; 3 Department of Psychology, Catholic University of Sacred Heart, Milan, 20123 Italy

**Keywords:** Health psychology, Health behavior, Hemodialysis, Kidney failure, Patient health engagement, Perceived stress, Treatment adherence

## Abstract

The present research examined the moderating effect of patient health engagement on the relationship between perceived stress and treatment adherence among patients with kidney failure undergoing hemodialysis. In this cross-sectional study, 184 patients from three hemodialysis outpatient clinics in Türkiye completed the Perceived Stress Scale, Patient Health Engagement Scale, and End-stage Renal Disease Adherence Questionnaire. Descriptive and inferential analyses preceded a moderation test using Hayes’ PROCESS macro (Model 1) with age and weekly dialysis frequency as covariates. Results supported H1, showing that higher perceived stress was associated with lower adherence (b =− 25.90, SE = 6.38, *p* < 0.001), and in line with H2, the stress × engagement interaction was significant (b = 4.29, SE = 1.24, *p* = 0.001, ΔR^2^ = 0.034), suggesting that engagement buffers the adverse impact of stress on adherence. Simple-slope analyses revealed that stress negatively predicted adherence at low (b = − 12.98, *p* = 0.001) and average engagement (b = − 7.09, *p* = 0.011), but not at high engagement (b = − 1.20, *p* = 0.697). These findings highlight patient health engagement as a protective resource that buffers stress-related non-adherence, suggesting that interventions to strengthen engagement may enhance adherence in hemodialysis care.

## Introduction

Kidney failure (KF), signifying the most severe form of chronic kidney disease (CKD), is characterized by a progressive, irreversible deterioration in kidney function [30], with hemodialysis standing as the foremost treatment modality [50, 53]. The rigorous demands of hemodialysis include strictly attending dialysis sessions, adhering to fluid and dietary restrictions, and following prescribed medication regimens [48]. Despite its life-sustaining significance, adherence remains suboptimal [6, 43], and as many as 30% to 86% of patients miss scheduled dialysis sessions [38]. Reports indicate dietary non-adherence rates ranging from 47.3% to 72.5%, fluid restriction non-adherence between 50% and 70.7% [55], and medication non-adherence near 32% [35]. Such non-adherence leads to poorer clinical outcomes, including higher hospitalization rates, aggravated disease course, and increased mortality [17]. Identifying factors influencing treatment adherence is critical to improving care and long-term health outcomes in this patient population.

Among the psychological determinants of adherence, perceived stress is a key factor shaping self-management behaviors in patients with chronic diseases [7, 13]. Perceived stress refers to individuals’ appraisal of life demands as exceeding their coping resources, thus guiding health-related behaviors by determining behavioral responses to illness management [16]. Stress can undermine cognitive functions critical for adherence, such as memory, attention, and decision-making, in the long term [59] and drive emotional dysregulation, leading to maladaptive coping strategies like avoidance, denial, and disengagement [5, 58]. In the context of hemodialysis, stress is related to the subjective experience arising from physical, psychological, social, and healthcare-related challenges,specifically, perceived stress is significant due to disease progression, treatment constraints, reduced daily and social functioning, and financial burdens [26]. Psychologically, patients undergoing hemodialysis often experience anxiety, depression, and a diminished sense of control over their health, while socially, disruptions to work, relationships, and daily routines further exacerbate stress [27, 46]. Elevated psychological stress often goes hand in hand with procrastination and treatment avoidance [26, 51], which can undermine optimal adherence [6]. For this reason, recognizing and addressing perceived stress is vital for improving patient well-being and ensuring long-term disease management.

While perceived stress has been consistently linked to lower adherence [2, 13], some research indicates that specific psychological resources may buffer its adverse impact [9]. In this regard, patient health engagement has been recognized as a nurturing factor in improving disease management among hemodialysis patients [39, 43]. Patient health engagement refers to the extent to which patients are cognitively, emotionally, and behaviorally involved in managing their health, encompassing aspects such as self-efficacy, motivation, and proactive participation in treatment decisions [24]. In particular, this concept is theorized in the form of the “Patient Health Engagement Model” (PHE model) [23], encompassing a broader, psychological continuum of cognitive (e.g., health information seeking), affective (e.g., emotional adjustment), and behavioral (e.g., health behavior change) involvement of a patient in managing their health. Highly engaged patients typically exhibit greater self-efficacy, more effective coping strategies, and stronger relationships with healthcare providers, all fostering improved adherence [23, 25]. Moreover, patient health engagement may promote adaptive responses to stress by enabling hemodialysis patients to more effectively handle the psychological challenges associated with chronic illness [3, 9].

Although it may appear reasonable to assume that highly engaged patients inherently adhere to prescribed regimens, this assumption may oversimplify the complex relationship between health engagement and adherence. While patient health engagement is a precursor to adherence [25], these two constructs are not synonymous [36]. Engagement encompasses a broader scope of involvement—from understanding the disease and maintaining motivation to collaborating with healthcare providers in a care partnership to optimize outcomes and enhance the care experience [36]. In contrast, adherence specifically denotes following prescribed treatment plans [15]. For instance, a person may be emotionally invested but struggle with consistent adherence to strict behavioral modifications due to perceived stress, whereas another person may adhere to treatment due to external pressures or established routines without genuine engagement, which can pose difficulties in sustaining long-term adherence [23, 25].

Engagement has been shown to buffer stress effects on self‐management [33], however, its role as a moderator of the perceived stress-adherence link in hemodialysis patients remains underexplored. Understanding how these opposing forces—stress impairing and engagement enhancing self-management—interact is crucial for designing targeted interventions [60]. To address these gaps, the present study examines the relationships among perceived stress, treatment adherence, and patient health engagement in a hemodialysis population, guided by the following research questions:

1. How does perceived stress affect treatment adherence behaviors among KF patients undergoing hemodialysis?
2. Does patient health engagement moderate the relationship between perceived stress and treatment adherence in this population?

### Hypotheses

Drawing from research on the adverse effects of psychological stress on health behaviors [13, 16] and the theoretical tenets of the Patient Health Engagement Model [23], we proposed the following hypotheses:

### H_1_:

 Perceived stress is negatively and significantly associated with treatment adherence levels of KF patients undergoing hemodialysis.

### H_2_:

 Patient health engagement moderates the relationship between perceived stress and treatment adherence behaviors in this population.

## Methods

### Research design

The current study employed a cross-sectional design, collecting data through structured and validated questionnaires administered to KF patients undergoing routine hemodialysis therapy.

### Setting and participants

A convenience sample of individuals undergoing hemodialysis was recruited from three outpatient hemodialysis clinics in Türkiye between February and November 2023. The first clinic, part of an academic tertiary-care hospital, regularly monitored 60 patients. The other two clinics, both within a private hospital, followed 195 and 90 patients, respectively. Participants were eligible for inclusion if they met the following criteria: (i) aged 18 years or older, (ii) undergoing routine hemodialysis for at least three months, (iii) not hospitalized at the time of recruitment, (iv) capable of independent self-care, (v) able to communicate in Turkish, and (vi) free from cognitive impairment or any psychiatric disorders as defined by the Diagnostic and Statistical Manual of Mental Disorders, Fifth Edition (DSM-V). Participants’ ability to engage in independent self-care was determined based on self-reported information collected during the recruitment process. Specifically, participants were asked whether they independently managed their daily self-care activities, including medication adherence, dietary restrictions, and fluid management, as part of their routine hemodialysis treatment. Additionally, the clinical staff involved in the hemodialysis units provided input to ensure that eligible participants did not require continuous assistance for self-care.

The sample size was calculated using G*Power 3.1.9.7 software [14] based on an anticipated effect size of the multivariable correlation, statistical power (1 − b), and α level, which were set as 0.15, 0.95, and 0.05, respectively. The chosen effect size corresponds to a small-to-moderate effect according to Cohen’s conventions [57], which indicates a meaningful association that, while not large, holds practical significance in understanding the relationship between the study variables [18]. Consequently, the required sample size was at least 107 participants. During recruitment, 345 potential participants were screened in three hemodialysis units. Patients undergoing routine hemodialysis for less than three months (*n* = 74), unable to perform self-care (*n* = 28), having cognitive impairment (*n* = 16), and refusing to participate (*n * = 43) were excluded. Finally, the study was completed with 184 participants.

### Measures

Data collection tools included sociodemographic and clinical characteristics, perceived stress, treatment adherence, and patient health engagement. Sociodemographic and clinical characteristics encompassed age, gender, education level, employment status, time since diagnosis, comorbidity profile, time since dialysis initiation and study enrollment, weekly dialysis frequency, session duration, and body mass index (BMI). Participants’ most recent laboratory parameters—hemoglobin, albumin, Kt/V, urea reduction ratio (URR%), calcium, phosphorus, and potassium levels—were extracted from their medical records. All laboratory values were obtained within the past three months to ensure relevance to their current health status.

Perceived stress was assessed using the 10-item Perceived Stress Scale (PSS-10), which evaluates stress levels in daily life and disease-related experiences [10]. The Turkish-validated version employs a 5-point Likert scale (0 = never to 4 = always) [12]. Factor analysis identified two dimensions: perceived insufficient self-efficacy and perceived stress/distress. The scale includes four reverse-scored items, with total scores ranging from 0 to 40, where higher scores indicate more significant perceived stress. Scores below 21 suggest effective stress management, scores between 21 and 26 indicate moderate stress management with potential difficulties, and scores of 27 or higher suggest significant challenges in coping with stress. Eskin et al.’s adaptation study reported good reliability (Cronbach’s α = 0.82, test–retest reliability = 0.88) [12]. In the present study, the scale demonstrated excellent internal consistency (Cronbach’s α = 0.96).

Treatment adherence was assessed using the End-Stage Renal Disease Adherence Questionnaire (ESRD-AQ) [32]. The original ESRD-AQ is a self-reported tool that comprises 46 items across five sections: general and history-related information (5 items), adherence to hemodialysis sessions (14 items), medication adherence (9 items), fluid restriction adherence (10 items), dietary adherence (8 items). Within these sections, the first section seeks general information on patients’ ESRD and history of renal replacement therapy, while the other four sections inquire about treatment adherence behaviors. Responses to this instrument incorporate a combination of Likert-scale items, multiple-choice questions, and yes/no response options. Total scores range from 0 to 1200, with higher scores indicating better adherence. The six Likert-type items (14, 17, 18, 26, 31, and 46) directly assess treatment adherence behaviors. The Turkish adaptation study reported item-total correlation coefficients between 0.48 and 0.80, demonstrating a strong relationship between individual items and the overall scale [40]. In the present study, item-total correlation coefficients ranged from 0.56 to 0.75, confirming good internal consistency.

Patient health engagement was assessed using the Patient Health Engagement Scale (PHE-s®), a five-item measure of individuals’ active involvement in healthcare management [24]. Based on the PHE model, the scale categorizes engagement into four phases along a psychological continuum, ranging from low (blackout, arousal) to high (adhesion, eudaimonic project). The PHE-s® employs a single-factor, ordinal structure with seven response options, allowing patients to position themselves at intermediate levels and reducing social desirability bias. The Turkish validation study reported an ordinal alpha of 0.80, with categorical principal component analysis confirming construct validity, as all factor loadings exceeded 62.8% [54]. In the present study, the scale demonstrated strong internal reliability (Cronbach’s α = 0.91).

### Ethical considerations and procedure

The study received ethics approval from Hacettepe University Non-interventional Clinical Studies Ethics Board) (Date: 29.11.2022, Approval Number: 2022/20-28) and adhered to the principles of the Declaration of Helsinki. Administrative permissions were obtained from the healthcare institutions where the study was conducted. Additionally, the original authors and copyright holders secured authorization to use the survey scales via email correspondence. Study procedures were explained verbally and in writing, and participants were explicitly informed of their right to withdraw without consequences. No names or identifying information were recorded on research instruments to ensure anonymity.

During data collection, researchers visited outpatient units, informed head nurses about the study’s objectives and procedures, and obtained a weekly list of patients. Eligible patients diagnosed with kidney failure undergoing weekday hemodialysis were then approached. Researchers verbally explained the study and screened patients based on the inclusion criteria. Those who met the requirements and agreed to participate provided written informed consent. The data collection process for each participant took approximately 20 minutes.

### Statistical analysis

All analyses were conducted using SPSS version 29.0 (SPSS Inc., Chicago, IL, USA). Descriptive statistics were computed for all study variables. Cronbach’s alpha coefficients were calculated to assess internal consistency of the instruments. The distribution of numeric variables was examined through means, standard deviations, skewness, and kurtosis. Continuous variables were described using mean ± standard deviation (SD) for normally distributed variables, or the median ± interquartile range (IQR) for non-normally distributed variables, with the IQR reported as the 25th and 75th percentiles. Independent samples t-tests and one-way ANOVAs were used to compare treatment adherence levels across participant subgroups. Post hoc comparisons were conducted using Tukey’s HSD test as Levene’s test confirmed homogeneity of variances (*p* > 0.05). Harman’s single-factor test was performed to examine potential common method bias due to self-reported data. Spearman’s rank correlation was used to explore associations among PSS-10, ESRD-AQ, and PHE-s® scores, given heteroscedasticity (*p* < 0.001) and the ordinal nature of the PHE-s®.

**Fig. 1:**
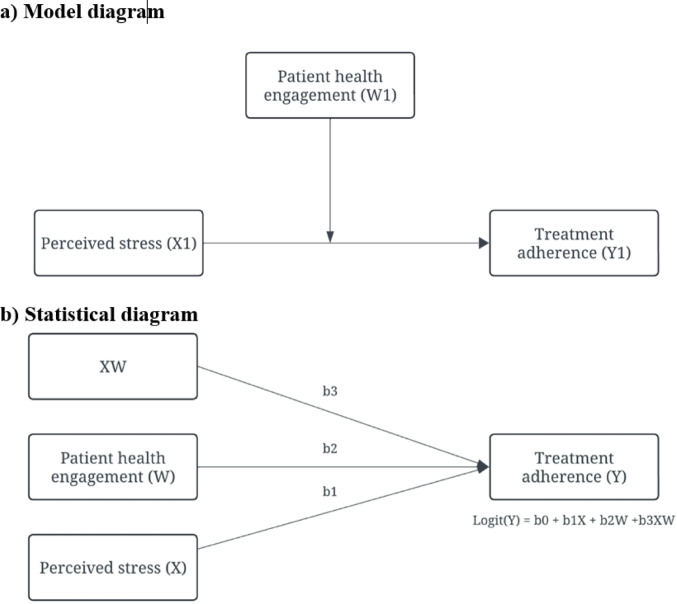
The moderation effect of patient health engagement on perceived stress in treatment adherence

To test the moderating role of patient health engagement in the relationship between perceived stress and treatment adherence, we used Hayes’ PROCESS macro (Model 1) with 5,000 bias-corrected bootstrap samples [28] (Fig. 1). Slope analysis was reported to interpret the conditional effects of the perceived stress on treatment adherence at different patient health engagement levels to facilitate the interpretation of the investigated moderating effects. A significant moderation effect was inferred if the interaction term was significant (*p* < 0.05) and the 95% bootstrap confidence interval excluded zero. A significance level of *p* < 0.05 was applied to all statistical analyses.

## Results

### Common method biases test

Before the data analysis, common method bias was controlled by conducting an exploratory factor analysis on all items of the key constructs (PSS-10, PHE-s®, and ESRD-AQ), examining the unrotated factor solution to identify the number of factors accounting for the overall variance. The Harman Single-Factor test [44] revealed three factors with eigenvalues exceeding one, with the first factor accounting for 31.8% of the total variance—well below the 50% threshold—indicating that common method variance is unlikely to significantly affect the study findings.

### Demographic and illness-related characteristics of participants

The sample consisted of 184 KF patients undergoing hemodialysis treatment. The socio-demographic data demonstrated an almost equal distribution between female and male patients, with an average age of 56.4 ± 15.47 years. One-third of the participants were high school graduates, and most (84.2%) were unemployed or retired. The prevalence of comorbidities was 93.5%, the duration of kidney failure diagnosis was 11.7 ± 8.55 years, and the time since dialysis initiation and study enrollment was 7.0 (3.0–11.0) years (Table 1).

**Table 1:** Sociodemographic and clinical characteristics of patients undergoing Hemodialysis in scores of treatment adherence (*N* = 184)

Characteristics	*n* (%)	Mean of ESRD-AQ (± SD)
Age (years)^a^		
18–39	28 (15.2)	969.6 ± 154.16
40–64	91 (49.5)	970.1 ± 168.04
65–85	65 (35.3)	889.23 ± 187.75^†^
*p*-value		0.012^*^
Gender^b^		
Female	93 (50.5)	932.8 ± 179.71
Male	91 (49.5)	950.3 ± 174.10
*p*-value		0.252
Education level^a^		
Below secondary school	66 (35.8)	924.6 ± 174.88
Secondary school	50 (27.2)	958.0 ± 194.46
High school and above	68 (37.0)	945.6 ± 165.65
*p*-value		0.587
Employment status^b^		
Employed	29 (15.8)	959.9 ± 192.42
Unemployed or retired	155 (84.2)	938.1 ± 174.05
*p*-value		0.551
Comorbidity existence^b^		
No	12 (6.5)	891.7 ± 212.22
Yes	172 (93.5)	944.9 ± 174.15
*p*-value		0.314
Length of each hemodialysis session *(hours)*^b^		
3.5	32 (17.4)	914.8 ± 163.24
4	152 (82.6)	947.0 ± 179.40
*p*-value		0.350
Number of hemodialysis sessions per week^b^		
2	24 (13.0)	992.7 ± 109.96
3	160 (87.0)	933.8 ± 183.65
*p*-value		0.033^*^

^a^ One-Way ANOVA, ^b^ Independent samples t-test

*Statistically significant

† Tukey’s HSD test indicated that treatment adherence is significantly lower compared to the 40–64 age group

The assessment of ESRD-AQ revealed no significant differences in treatment adherence levels based on gender, education, employment status, presence of comorbidities, time since diagnosis, time since dialysis initiation and study enrollment, or session duration. However, treatment adherence levels varied significantly with age and the number of hemodialysis sessions per week (*p* < 0.05; see Table 1). Table 2 presents the clinical and laboratory characteristics of the study population, including BMI and key blood parameters, to provide a descriptive overview of the study population.

**Table 2:** Clinical and laboratory parameters of patients undergoing Hemodialysis (*N* = 184)

Characteristics	*n* (%)	Mean ± SD/Median (range, IQR)
Body mass index (kg/m^2^)		25.1 ± 4.51
≤ 18	13 (7.1)	
18 to 25	78 (42.4)	
>25.1	93 (50.5)	
Hemoglobin (g/dL)		10.5 ± 1.51
≤ 10	68 (37.0)	
10.1 to 12	78 (42.4)	
>12	38 (20.6)	
Albumin (g/dL)		3.7 ± 0.48
≤ 3.5	37 (20.1)	
>3.5	147 (79.9)	
Kt/V (*n* = 162)		1.4 ± 0.27
≤ 1.2	41 (25.3)	
>1.2	121 (74.7)	
URR% (*n* = 168)		70.1 ± 8.67
≤ 65	37 (22.0)	
>65	131 (78.0)	
Calcium (mg/dL) (*n* = 164)		9.21 (6.06: 8.80–9.67, 0.87)
≤ 10	158 (87.8)	
>10	22 (12.2)	
Phosphorus *(mg/dL)*		5.1 ± 1.25
≤ 5.5	119 (64.7)	
> 5.5	65 (35.3)	
Potassium *(mmol/L)*		4.9 ± 0.73
≤ 5	111 (60.3)	
>5	73 (39.7)	

*SD*, Standard deviation, *Kt/V*, Measure of dialysis adequacy. *K*, clearance—the amount of urea your dialyzer can remove (liters/minute), *t*, time—the duration of treatment (minutes), *V*, volume—the amount of body fluid (liters), *URR%*, Urea reduction rate

### Descriptive characteristics and associations among perceived stress, treatment adherence, and patient health engagement scores

The mean PSS-10 score was 20.1 ± 8.54, with 50.5% of participants exceeding the threshold score of 21, indicating moderate stress. Specifically, 35.8% of patients were classified within stress level ‘C’, reflecting a sustained perception of threats and challenges that may negatively influence both their daily lives and disease progression. Additionally, 14.7% fell into stress level ‘B’, suggesting that while they exhibit some capacity for stress management, they may encounter difficulties in certain situations.

Participants’ adherence to hemodialysis treatment was evaluated using the ESRD-AQ by summing the scores of items 14, 17, 18, 26, 31, and 46. The mean overall adherence score was 941.4 ± 176.69. Findings indicated that 46.7% of patients demonstrated good adherence, 43.5% exhibited moderate adherence, and 9.8% had poor adherence to their treatment regimen. Adherence to specific aspects of hemodialysis care was also examined. The mean adherence scores were as follows: 288.1 ± 34.17 for hemodialysis sessions, 151.3 ± 46.29 for medication therapy, 126.4 ± 42.76 for fluid restriction, and 122.3 ± 56.23 for dietary recommendations.

The mean PHE® score was 4.4 ± 1.37. Notably, 8.2% of patients were in the blackout phase, while nearly half (45.1%) were in the arousal phase. Additionally, 36.4% were classified in the adhesion phase, and 10.3% had reached the eudaimonic project phase.

Table 3 provides the descriptive statistics for each observed variable, including mean, standard deviation, skewness, and kurtosis. All items satisfy the acceptable skewness and kurtosis thresholds (− 1 to 1), indicating that the data approximate a normal distribution. In addition, Table 4 summarizes the Spearman’s correlation coefficients among the study measures. Perceived stress showed significant inverse associations with patient health engagement (*r* = − 0.884, *p* < 0.001) and treatment adherence (*r* = − 0.704, *p* < 0.001). Conversely, patient health engagement and treatment adherence were strongly and positively correlated (*r* = 0.700, *p* < 0.001).

**Table 3:** Mean, standard deviation, skewness, and kurtosis of the study measures (*N* = 184)

Variables	Mean	Median	SD	Skewness (S.E.)	Kurtosis (S.E.)
PSS-10					
Item 1	2.21	2.00	0.99	0.26 (0.18)	−0.77 (0.35)
Item 2	2.05	2.00	1.19	0.21 (0.18)	−0.92 (0.35)
Item 3	2.29	2.00	0.79	0.27 (0.18)	−0.28 (0.35)
Item 4	1.67	2.00	0.97	−0.05 (0.18)	−0.91 (0.35)
Item 5	1.79	2.00	1.06	0.11 (0.18)	−0.69 (0.35)
Item 6	2.01	2.00	1.01	−0.05 (0.18)	−0.89 (0.35)
Item 7	1.83	2.00	0.83	−0.03 (0.18)	−0.92 (0.35)
Item 8	1.88	2.00	0.99	−0.09 (0.18)	−0.88 (0.35)
Item 9	2.16	2.00	0.87	0.12 (0.18)	−0.94 (0.35)
Item 10	2.17	2.00	1.14	0.03 (0.18)	−0.93 (0.35)
ESRD−AQ					
Item 14	288.04	300.00	34.17	−0.93 (0.18)	0.84 (0.35)
Item 17	167.39	200.00	48.71	−0.87 (0.18)	−0.09 (0.35)
Item 18	86.00	100.00	21.34	−0.79 (0.18)	0.60 (0.35)
Item 26	151.35	150.00	46.29	−0.51 (0.18)	−0.77 (0.35)
Item 31	122.28	150.00	56.23	−0.08 (0.18)	−0.62 (0.35)
Item 46	3.27	3.00	1.13	0.41 (0.18)	−0.93 (0.35)
PHE-s®					
Item 1	4.27	5.00	1.10	−0.75 (0.18)	−0.15 (0.35)
Item 2	4.46	5.00	1.51	0.30 (0.18)	0.94 (0.35)
Item 3	4.37	4.00	1.64	−0.18 (0.18)	−0.78 (0.35)
Item 4	4.17	5.00	1.78	−0.19 (0.18)	−0.75 (0.35)
Item 5	4.60	5.00	1.59	−0.19 (0.18)	−0.89 (0.35)

*SD*, Standard deviation; *SE*, Standard error; *PSS-10*, Perceived Stress Scale; *ESRD-AQ*, End-Stage Renal Disease Adherence Questionnaire, PHE-s® Patient Health Engagement Scale

**Table 4:** Correlations between perceived stress, treatment adherence, and patient engagement levels of patients undergoing Hemodialysis (*N* = 184)

Variables	Index	PSS-10	ESRD-AQ	PHE-s®
PSS-10	Spearman’s rho (ρ)	–		
	*p-*value	–		
ESRD-AQ	Spearman’s rho (ρ)	−0.704	–	
	*p-*value	< 0.001^*^	–	
PHE-s®	Spearman’s rho (ρ)	−0.884	0.700	–
	*p*-value	< 0.001^*^	< 0.001^*^	–

*PSS-10*, Perceived Stress Scale; *ESRD-AQ*, End-Stage Renal Disease Adherence Questionnaire, PHE-s®, Patient Health Engagement Scale

* Statistically significant

### Moderating role of patient health engagement on the association between perceived stress and treatment adherence

The moderation analysis, through Hayes’ PROCESS Model 1, tested the hypotheses while controlling for age and weekly hemodialysis frequency [28]. As shown in Table 5, consistent with H_1_, perceived stress was strongly and negatively associated with treatment adherence (b =–25.90, SE = 6.38, t =–4.06,  *p * < 0.001), indicating that higher stress corresponds to lower adherence. In support of H_2_, the stress × engagement interaction was significant (b = 4.29, SE = 1.24, t = 3.48, *p* = 0.001, ΔR^2^ = 0.034, F(1,178) = 12.09, p = 0.001), demonstrating that patient health engagement moderates the impact of perceived stress on treatment adherence. The overall model was significant (F(5, 178) = 36.63, *p* = 0.001) and explained 50.7% of the variance in adherence (R^2^ = 0.507).

**Table 5:** Results of the moderation analysis (*N* = 184)

Variables	b	SE	t	*p*-value	95% CI lower	95% CI upper
Constant	1352.468	209.569	6.453	< 0.001*	938.908	1766.029
Perceived stress (PSS-10)	–25.897	6.376	–4.061	< 0.001^*^	–38.480	–13.314
Patient health engagement (PHE-s®)	–43.091	32.849	–1.311	0.191	–107.917	21.733
Perceived stress x Patient health engagement	4.294	1.235	3.476	0.001^*^	1.856	6.732
Age	0.384	0.632	0.607	0.544	–0.864	1.633
Number of weekly HD sessions	–19.379	27.711	–0.699	0.485	–74.064	35.305

R^2^ = 0.507, ΔR² for interaction = 0.034, overall F(5, 178) = 36.63, *p* < 0.001

*b*, unstandardized regression; *SE*, standard error; *CI*, confidence interval; *PSS-10*, 10-Item Perceived Stress Scale; PHE-s®, Patient Health Engagement Scale; *HD*, hemodialysis

* Statistically significant

**Fig. 2:**
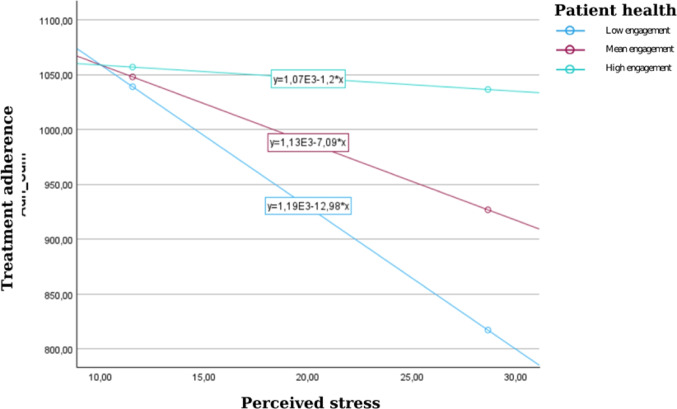
Slope analysis to interpret the moderating effect of patient health engagement on the association between perceived stress and treatment adherence

Probing the interaction revealed that perceived stress predicted poorer adherence at low (–1 SD) engagement (b = − 12.98, SE = 3.42, t = − 3.79, *p* = 0.001, 95% CI [–19.74, − 6.23]) and mean engagement (b = − 7.09, SE = 2.77, t = − 2.56, *p* = 0.011, 95% CI [–12.56, − 1.62]), but not at high (+ 1 SD) (b = − 1.20, SE = 3.07, t = − 0.39, *p* = 0.697, 95% CI [–7.25, 4.85]). These findings indicated that higher engagement buffers the negative effect of perceived stress on treatment adherence in patients undergoing hemodialysis, thus supporting H_2_ (Fig. 2).

## Discussion

The present study contributes to our understanding of how perceived stress may undermine adherence in patients undergoing hemodialysis while demonstrating the buffering role of patient health engagement. Consistent with prior work identifying stress as a key factor in non-adherence [61], our findings confirm that heightened stress perceptions are linked to poorer treatment adherence and that greater patient health engagement is associated with improved adherence. Most importantly, aligned with Hypothesis 1, higher perceived stress predicted significantly lower adherence, and in support of Hypothesis 2, patient health engagement moderated this relationship: engagement attenuated the negative impact of stress on adherence, even when controlling for age and weekly dialysis frequency. These results have clear implications for designing interventions that foster engagement to protect against stress-related non-adherence.

Our findings confirmed the negative association between high perceived stress levels and the participants’ ability to adhere to their treatment. These findings are supported by recent studies pointing out that the level of perceived stress experienced by individuals undergoing hemodialysis has been linked to decreased adherence to treatment regimens, including medication adherence and attendance at hemodialysis sessions [52, 62]. Evidence revealed that stress can impact patients’ coping mechanisms and ability to manage their condition effectively, which may lead to potential non-adherence issues [1]. In particular, perceived stress may disrupt executive functioning and emotion regulation [19, 31], which may potentially impede patients’ abilities to manage tasks ranging from fluid restrictions to medication schedules. Accordingly, patients experiencing stress might find it challenging to maintain a positive outlook and motivation to adhere to their treatment plan, which can ultimately affect their overall health outcomes [45]. From the standpoint of disease self-management literature [22, 23, 29], these results highlight the necessity of considering both psychosocial and clinical elements to improve long-term outcomes among individuals undergoing hemodialysis.

Furthermore, our findings revealed a significant negative association between perceived stress and patient health engagement. The chronic and intrusive nature of hemodialysis often leads to high levels of perceived stress, which can manifest as feelings of helplessness, anxiety, and emotional exhaustion [27]. Perceived stress may directly impair health engagement by depleting the cognitive and emotional resources necessary for patients to manage their condition effectively [9]. At the behavioral level, stress can lead to non-adherence to dialysis sessions, dietary restrictions, and medication regimens [8], as patients may prioritize immediate emotional relief over long-term health goals. Additionally, stress exacerbates psychological comorbidities such as depression and anxiety [11, 20], which may further reduce patients’ motivation and self-efficacy [21] — both considered key drivers of health engagement [36]. Additionally, perceived stress can undermine patient engagement by affecting individuals’ mental well-being and ability to participate in their care actively [56]. Studies have shown that stress-related emotional distress can hinder effective communication with healthcare providers and may result in missed opportunities for shared decision-making and personalized care planning [34, 47]. These shreds of evidence suggest that addressing stress as a psychological factor is essential in promoting patient engagement and improving treatment outcomes in individuals undergoing hemodialysis.

One of the most compelling findings is that patient health engagement moderated the perceived stress–adherence link, buffering the adverse impact of perceived stress on treatment adherence. This underscores patient health engagement as a protective mechanism, consistent with theoretical frameworks defining it across cognitive (e.g., knowledge acquisition), emotional (e.g., self-efficacy, motivation), and behavioral (e.g., active collaboration with healthcare providers) processes that empower patients to cope with the ongoing demands of treatment [23]. By fostering collaboration and co-responsibility, higher engagement also bolsters resilience to perceived stress, reducing the likelihood of avoidance-based coping or withdrawal from recommended behaviors [19]. Thus, engagement functions not merely as an outcome but as an active resource that shapes how stress is internalized and transformed into health behaviors [41, 42, 49, 61]. These insights deepen our understanding of adherence in hemodialysis, emphasizing that promoting engagement may recalibrate patients’ appraisal of illness-related stressors and enhance their capacity to maintain consistent treatment behaviors despite the emotional and physical burdens of chronic illness.

Our findings suggested that most patients fall into the lower engagement categories (8.2% in blackout and 45.1% in arousal), suggesting that more than half of the sample is grappling with emotional or informational barriers that hinder full participation in their care. According to the PHE model, individuals in the blackout phase often experience feelings of overwhelm or denial [4, 23], whereas those in the arousal phase display some recognition of their health needs yet remain hesitant or only partially activated, possibly due to lingering psychological distress or insufficient knowledge of CKD management [19, 23]. By contrast, 36.4% in the adhesion phase and 10.3% in the eudaimonic project phase signify more advanced engagement levels in which patients display greater self-efficacy, collaborative attitudes with healthcare providers, and an intrinsic drive to optimize their well-being [37]. Such skewed distribution underscores the need for tailored interventions that help patients in the earlier stages of the disease build requisite coping skills, self-efficacy, and emotional readiness to progress toward more sustained, autonomous disease management.

Although our findings add a crucial piece to the literature on psychosocial factors in CKD, several considerations limit the scope of the conclusions. First, the cross-sectional design precludes establishing a definitive causal chain among stress, engagement, and adherence; future longitudinal research could clarify whether targeted interventions to reduce stress or enhance patient health engagement result in meaningful improvements in adherence patterns. Second, the use of self-reported adherence measures, while standard in adherence research, may be subject to recall bias and social desirability effects, potentially leading to overestimation or underestimation of actual behaviors. Future studies should consider triangulating self-reported data with objective indicators such as interdialytic weight gain, serum phosphorus levels, or pharmacy refill records to strengthen the validity of adherence assessment. Third, the study did not account for variations in treatment protocols or institutional practices across dialysis facilities, which may have influenced treatment adherence. Fourth, participants were drawn from three clinics within a single geographical region, limiting generalizability to populations with different cultural, healthcare, or socioeconomic contexts. Broader, multicenter investigations could address potential variability in stress appraisal, engagement levels, and adherence behaviors. Lastly, several practical and psychosocial factors may have contributed to this population’s relatively high refusal rate. Dialysis treatment itself can be physically and emotionally burdensome, often leaving patients too fatigued or demotivated to participate in research activities. Additionally, rigid dialysis schedules may restrict the time and energy patients have to complete study procedures.

## Conclusion

The present study confirms that perceived stress undermines treatment adherence among hemodialysis patients and demonstrates that patient health engagement buffers this adverse effect. In particular, higher engagement—manifested by active collaboration with healthcare providers, enhanced self-efficacy, and emotional readiness—attenuates the negative relationship between stress and adherence, although stress still exerts some impact at lower engagement levels. This moderation highlights the multifactorial nature of adherence and underscores the importance of psychosocial resources in sustaining self-management.

From a clinical standpoint, these findings support the routine assessment of both perceived stress and engagement in nephrology settings. Screening for perceived stress using valid and reliable measures should become a standard practice to promptly identify vulnerable individuals before non-adherence leads to adverse outcomes, such as increased hospitalization rates and disease progression. Interventions aimed at bolstering engagement—such as structured educational programs, motivational interviewing, and consistent involvement of patients in shared decision-making—could moderate the detrimental effects of stress on adherence and foster more sustained health behaviors. These strategies might be especially beneficial for those at an early stage of dialysis treatment as they adjust to the regimen and face acute stressors related to frequent sessions, dietary changes, and lifestyle constraints.

## Data Availability

Due to privacy and ethical restrictions, the datasets generated and/or analyzed during the current study are not publicly available, but they are available from the corresponding author upon reasonable request.
